# Perspective of healthcare professionals on barriers and facilitators in exploring end-of-life care preferences of patients with pulmonary fibrosis: A qualitative study

**DOI:** 10.1371/journal.pone.0338624

**Published:** 2025-12-12

**Authors:** Lian Trapman, Lea M Dijksman, Jan C. Grutters, Saskia C.C.M. Teunissen, Everlien de Graaf

**Affiliations:** 1 Interstitial lung diseases Center of Excellence, member of European Reference Network-Lung, St Antonius Hospital, Nieuwegein, the Netherlands; 2 Department of Value-Based Healthcare, St Antonius Hospital, Nieuwegein, The Netherlands; 3 Division of Heart and Lungs, University Medical Center Utrecht, Utrecht, The Netherlands; 4 Center of Expertise Palliative Care Utrecht, Julius Center for Healthcare Sciences and Primary Care, Department of General Practice, University Medical Center Utrecht, Utrecht, The Netherlands; Government Villupuram Medical College and Hospital, INDIA

## Abstract

**Background:**

Progressive pulmonary fibrosis is a lethal disease with a survival of 3–5 years with optimal medication treatment. Palliative care and advance care planning are therefore receiving increasing attention in the literature. However, structural implementation in clinical practice is still lacking. The aim of this study was to explore the needs, facilitators, and barriers for communication on the topic of end-of-life preferences of patients from the perspective of healthcare professionals.

**Methods:**

A generic qualitative study was performed with focus groups and individual semi structured interviews with healthcare professionals. Data collection and analysis were performed iteratively. A thematic analysis was performed, following the methods of Braun and Clarke.

**Results:**

Three focus groups and seven individual semi-structured interviews were conducted. Three themes were generated: (1) a lack of vision on palliative care, resulting in different approaches within the same clinic and showing the need for optimization of collaboration; (2) the importance of a learning-driven environment to support healthcare professionals skills and knowledge; and (3), the central role of the individual professional in developing skills and knowledge.

**Conclusions/discussion:**

This study underscores the importance of behavioral and organizational change in palliative care to optimize conversations exploring values, preferences, and needs for end-of-life care for patients with pulmonary fibrosis. Leveraging the shared motivation of healthcare professionals to provide optimal care, integrating these findings into training and coaching programs can further enhance patient-centered approach in palliative care.

## Introduction

Pulmonary fibrosis (PF) is a rare, progressive, and chronic lung disease [1–3]. The prognosis varies considerably, influenced by factors such as timing of diagnosis and patient’s tolerability of medications.

While survival without medication typically ranges from three to five years, the use of antifibrotics has been linked to an extension of this timeframe, by slowing the decline in Forced Vital Capacity of the lung function by 50% [4,5].

Given this limited life expectancy, a proactive and structured palliative care approach could offer significant benefits. This approach aims to enhance quality of life, alleviate physical, psychological, social, and existential distress, and support patients and their families [6]. The guideline outlined by the European Respiratory Society (ERS) for palliative care in chronic obstructive pulmonary disease and interstitial lung diseases (ILD), including PF, underscores the importance of integrating palliative care interventions into overall management of PF [7]. These interventions should be multidisciplinary and holistic, addressing the personal and diverse needs of both patients and their families.

In general, palliative care interventions consist of multiple elements, including advance care planning, multidimensional symptom management, patient education, and supporting informal caregivers [8]. Advance care planning is a proactive, structured, and ongoing process of attending to patients’ values, wishes, and needs regarding life, care, treatment, and end-of-life preferences [9]. While its importance has been established in oncology, its implementation in PF care remains limited [10,11].

The implementation of advance care planning is receiving increasing attention and is starting to be adopted across a wide variety of medical specialties. However, this remains challenging. A recent review examined barriers and facilitators perceived by hospital clinicians when discussing patients’ values, wishes, and needs. The study found that these discussions primarily focus on treatment preferences and limitations. Identified barriers included professional attitude, perceptions of patients and their loved ones, clinicians’ human aspects, interprofessional collaboration, and contextual factors. Furthermore, an uncertain prognosis reduced healthcare professionals (HCPs) confidence in initiating these conversations [12].

Veigh et al. also highlighted that this uncertainty impacted the timely provision of palliative care [13]. A recent study in the United States found that, while most HCP’s provided palliative care and felt comfortable discussing palliative care patients’ needs, they faced several barriers. These included limited local access, lack of systematic tools for symptom assessment, and uncertainty about the optimal timing for the initiation of palliative care [14].

Previous studies with idiopathic pulmonary fibrosis patients showed that while patients are willing to talk about values, wishes, and needs at the end of life, they believe the initiative should come from the HCP. Patients also mentioned a lack of information about future expectations [15–17].

Although patients expressed a need for discussions about end-of-life issues, HCPs seemed to sub-optimally implement this. The objective of this study was to identify the barriers and facilitators that HCPs encounter when exploring values, preferences, and needs for end-of-life care with PF patients. This represents a first step towards improving the integration of palliative care in this patient population.

## Methods

### Design

An explorative generic qualitative study with healthcare professionals (HCPs) working in a Dutch hospital was performed from 2021 to 2022. This study was set-up from an interpretivist/constructivist paradigm, assuming that individual construct their own social realities through interaction and experience [18]. Given our research focus, this paradigm allowed us to approach the topic in a way that prioritizes understanding the participants’ perspectives within their specific context. For this report, standards for reporting qualitative research- guidelines were used [19].

### Characteristics and reflexivity

Interviews were conducted by two separate interviewers (JH and LT), focus groups (FG) were led by LT and supported by JH. JH is an experienced nurse in pulmonology and was trained on the job for interviewing. LT is an experienced clinical academic nurse in pulmonology, trained in collecting qualitative data through semi-structured individual interviews, and FGs. Parallel to this study, LT was trained in palliative care. Interviewers were familiar with the aim of this study and had a working relationship with all HCPs who were interviewed. Given the existing relation between interviewer and interviewees, there was a risk of “going native”, in other words, potentially blurring the line between researcher and participant [20]. Interviewers were aware of this and memos were written by them to reflect on the influence they might have had on the results. Additionally, they reflected on this during research meetings. These measures were undertaken to manage potential biases and ensure that findings reflected participants’ perspectives rather than those of the interviewers.

While analyzing data and discussing themes, the entire, multidisciplinary research team was involved. Coding was done by LT and LD, with the latter having no experience in providing care. A senior qualitative researcher EdG supervised the data analysis process. All analyses, including the identification of categories and themes, were subsequently discussed within the entire research team.

### Context

This study is performed in a referral center for interstitial lung diseases. The HCPs are specialized in interstitial lung diseases. The care in this center is delivered by an interdisciplinary team with specialized pulmonologists and nurses. A specialist palliative care team is available, who can support palliative care in inpatient and outpatient clinics.

### Study population

All HCPs working in active inpatient and outpatient care in this department were eligible for this study. Starting June 1^st^, 2021, HCPs were informed about the overall aim of this study and asked to participate via e-mail, and face-to-face in informal meetings. Participants were selected through convenience sampling, based on voluntary participation, with efforts to include a range of roles and experience levels within the multi-professional team.

After fifteen interviews, no new themes or insights were identified. An additional interview with a HCP of differing characteristics and perspective also revealed no new themes. Recruitment ended at March 10^th^ 2022.

### Ethics

This study followed the Declaration of Helsinki [21], Good Clinical Practice Guidelines [22] and the General Data Protection Regulation [23]. This study is not subject to the ‘Medical Research Involving Human Subject Act (WMO) as it does not concern medical scientific research, and participants are not subject to procedures or required to follow rules of behavior. The study protocol was approved by the Medical research Ethics Committees United (MEC-U W21.018). All participants gave verbal informed consent for the use of their data.

Participants’ consent and interview records were documented and securely stored in REDCAP (www.project-redcap.org) and pseudonymized by giving them a code number in transcription procedures. Only LT and JH had access to recordings, transcripts, and the pseudonymization document on two different hard disks.

### Data collection

All interviews were conducted between June 2021 and March 2022. The combination of FGs and individual interviews was chosen to first get insight into relevant topics as discussed between professionals. These topics were then explored in greater depth during subsequent individual semi-structured interviews. This sequential approach allowed us to identify shared themes through group interaction, and then examine personal experiences and nuances that may not surface in a group setting. The use of both methods provided complementary perspectives and enriched the overall understanding of the research topic.

HCPs only participated once, either in an interview or a FG, based on personal preference. In FGs, all disciplines were mixed together. During interviews, only the interviewer and the participant were present. Due to COVID-19 regulations one FG was performed online, using Pexip (www.pexip.com). Other FGs and interviews were performed face-to-face in a conference room in the hospital.

Data collection and analysis were alternated, with new questions that raised during the analysis being included in the interview guide for subsequent interviews.

Two interview guides were developed (S1 Appendix), for FGs and semi-structured interviews, based on literature and the research team’s experiences. All questions were open ended. The interview guide for FGs was designed to initiate discussions among HCPs, encouraging them to identify and discuss important themes. FGs were conducted prior to individual interviews and were used to optimize the interview guide for individual interviews. See interview guides for more details,

### Data processing

FGs and all interviews were digitally audio recorded, transcribed verbatim, and then anonymized. Transcription and anonymization were performed by working students. Subsequently, transcripts were uploaded into the Atlas.ti 9 application together with memos. From that point onward all data was processed in Atlas.ti 9.

### Data analysis

A thematic analysis was performed following the six phases described by Braun and Clarke [24], here shown in *Italics*. Two researchers (LD, LT) first *familiarized* themselves with the data by reading and re-reading the transcripts. *Initial coding* was performed independently and inductively on two transcripts in Atlas.ti (LT and LD), using open and detailed coding while staying close to participants’ wording.

Based on this initial coding, a preliminary coding framework was developed and applied to the subsequent transcripts (LT, LD). Each interview was discussed to reach shared understanding and refine interpretation.

Codes were then organized into meaningful clusters, first manually and later using Atlas.ti functions. These clusters formed the basis for developing *potential themes* and subthemes, which were *reviewed*, *refined*, and *named* through discussion with the multi-professional team.

Theme development was approached as an active and interpretative process, consistent with reflexive thematic analysis, rather than as a passive emergence from the data.

Descriptive statistics were used to present characteristics of the participants

### Trustworthiness

To enhance **credibility**, memos were written after each FG and individual interview, during transcription, and throughout the data analysis process. Member checking during interviews helped ensure that participants’ perspectives were accurately captured.

To ensure **dependability,** an audit trail was maintained throughout the whole process of data collection and analysis, documenting all decisions made during the study. Coding and theme development were discussed and reviewed by multiple researchers to strengthen consistency.

**Transferability** was supported by providing detailed descriptions of participants’ roles, experiences, and the context in which the study took place, allowing us to assess the applicability of findings to other settings.

**Confirmability** was promoted through the use of reflexive memos and regular team discussions, which helped minimize interviewers’ and researchers’ biases and ensure that interpretations were grounded in the data.

## Results

In total, 18 HCPs agreed to participate. Later, two HCPs withdrew consent to participate because of a lack of time, resulting in a final sample of 16 HCPs. Characteristics are shown in Table 1. Mirroring the team characteristics, the majority of the participants are female and work in the outpatient clinic less than ten years.

**Table 1:** Participants characteristics.

*Items*		*N (%)*
Participants		16 (100%)
Female		12 (75%)
Age	20-30	5 (31%)
	30-40	7 (44%)
	>40	4 (25%)
Department*	Inpatient ward	8 (50%)
	Outpatient ward	13 (81%)
Disciplines	Nurses	10 (63%)
	Physicians	4 (25%)
	Other disciplines	2 (12%)
Educational level (ISCED)*	4	5 (31%)
	5	2 (13%)
	6	5 (31%)
	≥7	4 (25%)
Working experience (years)	0-5	9 (56%)
	5-10	6 (38%)
	10-15	0 (0%)
	15-20	1 (6%)

*Participants can be working in both inpatient and outpatient ward; **ISCED = International Standard Classification of Education (ISCED) [25]: *4: Post-secondary non-tertiary education; 5: Short-cycle tertiary education; 6: Bachelor’s or equivalent level; 7: Master’s or equivalent level; 8: Doctoral degree or equivalent level.*

In total, three FG, each with three HCPs, were carried out, followed by seven interviews. The mean durations of the FGs and interviews were 97 (50–120) minutes and 45 (36–50) minutes, respectively.

Participants expressed a strong motivation to discuss end-of-life issues with patients, recognizing this as a crucial aspect of their role, especially with patients facing life-limiting progressive diseases. Although most questions focused on personal reflections regarding their role in patient communication, all participants also addressed broader organizational aspects of care. Common barriers, such as time constraints and location, emerged early in nearly all sessions and were considered fundamental prerequisites. Yet, even with ample time and ideal consultation settings, other obstacles would persist.

While these elements are primarily viewed as barriers, their inherent duality allows them to also act as facilitators depending on contextual factors; therefore, they are presented as thematic elements rather than categorized strictly as barriers or facilitators. Three main themes were generated from the data: vision on palliative care, the importance of a learning-driven environment, and the role of the individual professional.

### Lack of a shared vision on palliative care

Unlike disease-specific treatment, palliative care lacks a unified approach. Consequently, each healthcare professional (HCP) may adopt their own interpretation of palliative care, leading to variability in services and care provided. This diversity is reflected in three subthemes: use of language, collaboration within the team, and collaborations outside the team.

### Use of language

Participants defined the onset of the palliative phase in diverse terms, including “no longer curable,” “when progression is noted”, “final phase of life,” “upon a terminal diagnosis,” and “a hybrid event”. This variability extends to discussions on personalized and patient centered care. While some aim to align care with patients’ wishes and values, others may center care around the patient, without actively involving them in decision making. HCPs admit to not verifying their assumptions with the patient and their partner, tending instead to project their own feelings and values.

*‘Then we often notice that people haven’t heard the term “palliative care” very often, or that it has never really been brought up.’ (participant 2)*

### Collaboration within the team

The lack of a unified understanding, and clear definitions regarding palliative care, results in confusion about responsibilities and task allocation within the team. Roles of nurses and physicians often remain ambiguous, leading to inconsistent patient care. While some HCPs proactively explore and manage their patients’ palliative care needs, others overlook necessary discussions on palliative care. This inconsistency is particularly pronounced when patients transition between specialists, a situation frequently encountered with specialists on fixed-term contracts. Although one participant has a positive experience and saw potential benefits in this approach, the prevailing lack of clarity undermines consistent care delivery.

*‘It’s important to do it as a team: but that also means that the team needs to be on the same page... That’s why I find “palliative [treatment]” a difficult term to convey to patients. That’s why I prefer using symptom-focused to differentiate it from other treatments.’ (participant 1)*

Few participants suggested that established protocols and guidelines could enhance inter-professional collaboration in palliative care. However, they also explained that current palliative guidelines are not always applied by all HCPs.

### Collaboration beyond the team

One FG emphasized the importance of systematic documentation strategies to enhance collaboration among HCPs within the hospital. Effective communication between inpatient and outpatient clinics is crucial, sharing information on outcomes and underlying values and rationales, to ensure proper continuity and follow up. However, other participants raised concerns about the appropriate level of detail for documentation and how to discern what is critical to record.

The role of the Palliative care Advice Team (PAT), a specialized multi-professional palliative care team, was discussed in one FG and two interviews. Participants suggested that the PAT’s task should be to provide advice and coaching to ILD HCPs, rather than assuming direct care responsibilities or communicating directly with patients. This recommendation stems from the PAT’s limited knowledge of ILD and the belief that palliative care is integrated into the ILD care. However, the same interviewees also mentioned that the PAT is consulted excessively due to relative inexperience of trainee doctors and novice nurses in the inpatient ward. Additionally, during FG, some participants expressed unawareness of the PAT’s consulting role for the outpatient clinic and advocated for its increased involvement.

*‘I think that when having such a conversation about approaching end-of-life, you need to understand what the disease is, what the impact of the disease is, psychologically, socially, spiritually. We were also just talking about continuity [of HCPs]; I don’t think they [the Palliative advisory team] are the right people. I think they might know which elements should be addressed in the conversation… They can be coaching, but shouldn’t lead the conversation. So really advisory.’ (participant 13)*

Participants mentioned a wide range of perspectives on transmural collaboration, leading to varied approaches, and different levels of engagement with general practitioners (GP). Communication methods differ between HCPs, some prefer direct telephone communication for each patient, while others opt for letters. Nurses often maintain communication with primary care providers. Most pulmonologists continue to communicate with patients and/or their families, even after coordination of care has transitioned to the GP. Participants frequently attributed this ongoing contact to the perceived lack of ILD knowledge among primary care provider and to a personal desire to maintain a humane, personal connection.

*‘I think we only reach out [to contact the General practitioner] in the final stage [when a patient is dying]. Then we briefly mention it in the letter. Sometimes we call. But I think we’re a bit late with that as well. But the question is, if you [nurses] do it earlier, is that sufficient or not?’ (participant 9)*

### Learning-driven environment

Participants state that there exists a shared learning environment for acquiring knowledge and skills related to disease specific care. Within this environment, participants expressed confidence in the abundance of physical and medical knowledge, as well as in the ongoing development and dissemination of new insights. However, this shared learning-driven environment appears to be lacking when it involves palliative care and non-physical concerns and needs. In these areas, it depends on individual HCPs and his or her learning approach. Participants highlighted that currently, professional development relies heavily on individual initiative. While some participants reflect on their conversations and seek to learn from their mistakes, few mention peer to peer coaching, or group supervision as potential interventions.

*’I do reflect on that myself. I don’t really do it with the patient… If they mention something about the conversation, I will respond to this.’ (participant 13)*

Findings indicate there appears to be a noticeable gap in the development of relevant knowledge and skills when it comes to palliative care and end-of-life conversations. This gap is compounded by the absence of a structured approach to facilitate the exchange of this knowledge and skills among HCPs. Trainings, workshops and a culture of feedback are lacking, as evidenced by the knowledge and skills mentioned by participants.

*‘I lack knowledge and experience to effectively guide individuals from other cultures in the palliative phase.’ (participant from FG 3- from the wall: ‘not satisfied with..’)*

Participants explicitly mentioned the pivotal role of knowledge in initiating and conducting end-of-life conversations. They noted that the required knowledge varies depending on the HCPs profession and their responsibility. While physical symptoms are explicitly discussed, there is a notable absence of consideration for psychological, social and spiritual concerns such as coping mechanisms, loneliness, and grief. Despite expressing a desire to see patients as whole individuals, participants predominantly focused on the physical dimension. A multidimensional approach is only mentioned by HCPs with specialized education in pulmonology or mental healthcare.

*‘Of course, there are those questions you can ask from the Diamant model [Ars Moriendi model* [26]], *for example, which I don’t always have laid out on the table, but they are in my head.’ (participant 15)*

*‘I do believe that stress has a significant impact on someone’s illness, as well as their perception of illness. Perhaps that should be higher on the list of things we should address than medication for some people.’ (participant 11)*

Besides the expansion of knowledge, skills are also necessary to put this knowledge into practice. Almost all participants mentioned the importance of communication skills alongside care and treatment skills. However, there is a range of confidence levels among participants, ranging between completely confident to not confident enough and maybe avoiding discussions about end-of-life. Others felt generally confident but acknowledged the need for additional training.

*’Am I causing them more sorrow than necessary? ‘ (participant 12)*

Furthermore, participants identify prerequisites for effective palliative care and end-of-life conversations, including adequate time, appropriate patient information, and suitable locations. While these prerequisites are recognized by most participants, there is a lack of action taken to optimize them.

*‘So I’ve actually stopped doing that [giving out brochures]… Because I don’t find the information helpful. At one point, we received a brochure. It again had a sentence that was incorrect. And we have a very simplistic brochure... I really think it’s a baby brochure. You should also take your patient somewhat seriously. I’ve kind of stopped giving everything out, unless people ask for it themselves.’ (participant 4)*

### The individual professional

Besides the organizational and collaborative part of care, the individual professional influences conversations and interactions with the patient and his or her partner about end-of-life. This is reflected in two sub-themes: professional and personal experiences, and tailoring to the patient.

### Professional and personal experiences

Experiences of HCPs directly impact the care they deliver. Positive professional experiences facilitate effective end-of-life communication and increase confidence. Conversely, negative experiences may breed hesitation. Personal experiences also play a pivotal role, often serving as a source of motivation. HCPs reflect on their own experiences of having been a patient themselves or on experiences involving loved ones who required palliative care.

*‘It also touches on personal experiences, for example, my father with whom I experience the last phase… But it can also be inhibiting, and therein it’s also very important that you know yourself very well. ‘ (participant 15)*

However, a few participants noted that personal experiences can sometimes cloud judgment, and impede end-of-life conversations due to emotional involvement.

Moreover, while experience in end-of-life conversations is undoubtedly valuable, it is not universally perceived as the sole essential factor. Some participants state that possessing ‘guts’ and ‘trust on their skills’ suffice to address and follow up on end-of-life topics with patients.

*‘When I compare it to five years ago when I just started on the ward, I found it much more difficult back then. You think: oh, you want to discuss the topic of palliative care, but then you wonder if it’s already time. You doubt yourself. I don’t really have that anymore now… And I find it much easier to broach the subject… It’s through that experience that you know better to ask the right questions. ‘ (participant 3)*

### Tailoring to the patient

To ensure appropriate care for all patients, care needs to be tailored to the coping process and needs of individual patients. Therefore, the HCP needs to, for example, involve patients and partners in development of a care plan, and check preconceptions with the patient. One-size-fits-all care does not exist, and the fact that palliative care has a personalized character is mentioned by almost every participant. However, HCPs admitted that they do not always verify their assumptions with patients, and they appear to struggle when patients have different coping strategies or hold values and norms that differ from their own. Part of tailoring care to the patient involves considering intercultural differences and health literacy, however this was mentioned by only a few participants.

*‘That [to avoid disturbing the patient and their loved ones] is why I sometimes don’t have a conversation. Even though we’re still following a code B [limited treatment; no reanimation and intubation] I estimate it won’t last longer than a week. Especially the time for them together… and now that I’m saying this, I think, maybe you should just ask instead of assuming that it’s desired for them to be together.‘ (participant 14)*

## Discussion

This qualitative study aimed to identify barriers and facilitators that healthcare professionals (HCPs) from a tertiary ILD clinic encounter in exploring values, preferences, and needs for end-of-life care with PF patients.

Three interconnected themes were generated, each encompassing a range of barriers and facilitators: vision on palliative care, the learning environment, and the individual professional’s role. A shared vision on palliative care ensures a unified approach, but its absence leads to varied interpretations and inconsistent care. While disease-related knowledge is well-supported, palliative care education is lacking, resulting in insufficient skills for optimal care. Ultimately, the quality of care hinges on the individual professional’s interest and experience in palliative care.

Current literature offers various definitions of vision, all emphasizing an ideal future goal. An implemented shared vision on palliative care can serve as an ‘inner mental voice’, guiding behavior [27]. It provides orientation and meaning, helping leaders and teams focus their efforts and drive transformation of practice [28]. In contrast, this study highlights that the absence of a vision for palliative care leads to suboptimal collaboration and hindered professional development. Once the vision has been implemented and the corresponding roles are clear, professionals can align with it—both individually and as a team.

Assigned roles require specific knowledge and skills, supported by a professional environment that fosters growth. Conditions for professional development align with the themes identified in our study: organizational culture shapes these conditions, facilitates professionals, and the environment should be supportive [29]. Inter-professional communication fosters informal learning and assists professionals identifying their learning goals. Inter-professional education can enhance collaboration and patient care, but its effectiveness compared to other training methods remains unclear [30]. HCPs have a responsibility to remain up-to-date and continuously improve their skills by engaging in continuing professional development (CPD) [29,31].

In the Netherlands, CPD is voluntary and depends on individual motivation. This study reveals a significant gap in palliative care knowledge, both practical and emotional. While participants were confident in their communication skills, it is unclear whether they recognized these gaps. Without such awareness, voluntary education may fall short.

Ultimately, even with a clear vision and education, the quality of care in the consultation room depends on the individual professional. Patient-centered care, a common concept in healthcare, requires specific skills and knowledge. Various models aim to improve individual outcomes through shared elements such as a holistic approach, shared decision-making, empathy, active listening, alignment with patient values, family involvement, and education [32,33].While participants referred to patient-centered or personalized care, not all core elements were reflected in the interviews, leaving the approach only partially patient-centered. Education and coaching may support HCPs to reflect on and improve their practices.

A recent systematic review on barriers and facilitators of conversations about patients’ values, wishes, and needs for palliative hospital care identified similar challenges: lack of knowledge and skills, prognosis uncertainty, and collaboration [12]. It also highlighted the emotional and psychological burden on HCPs during these conversations, a topic scarcely mentioned by participants in this study.

**Fig 1:**
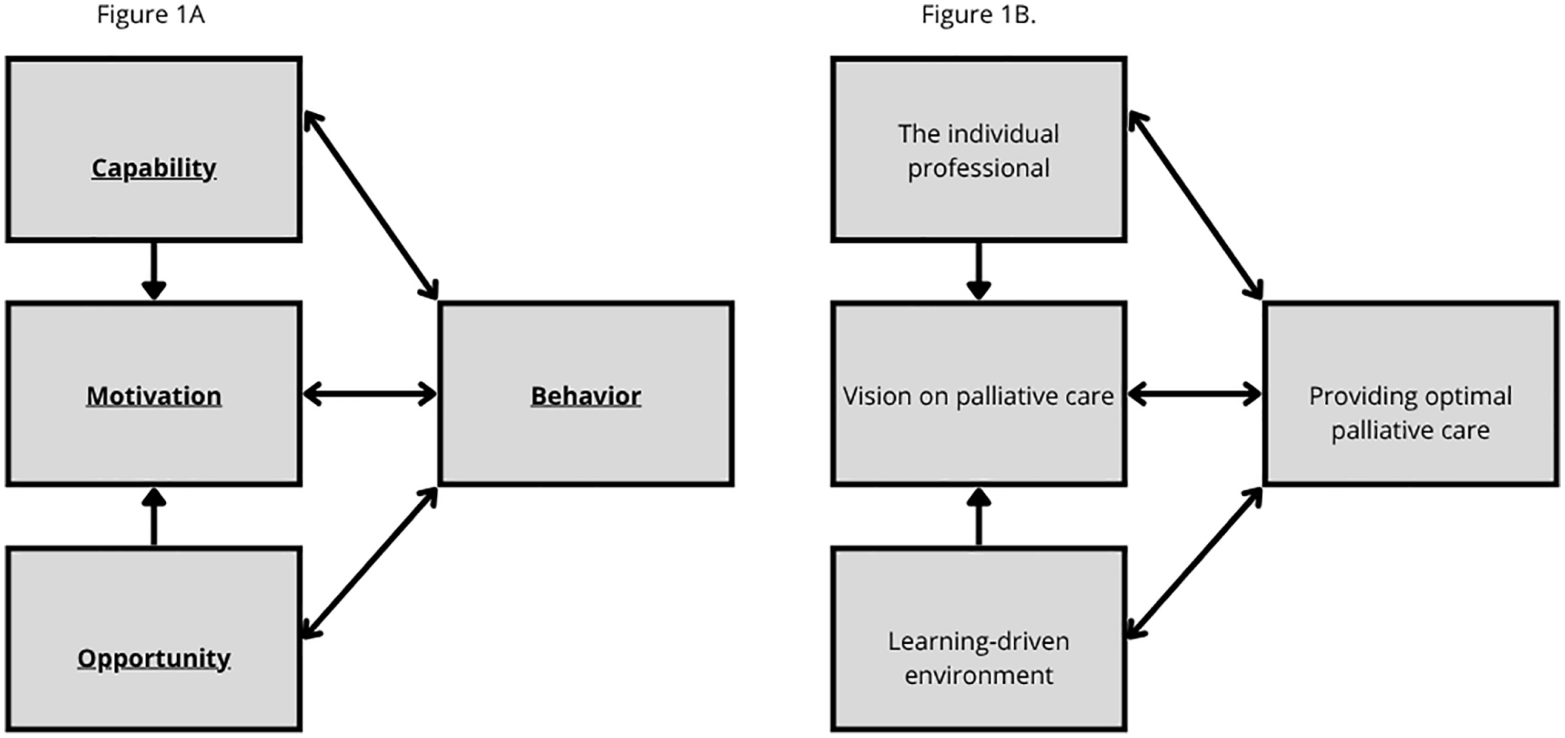
A. The original COM-B model. B. showing the relationship between the themes in this study and the behavior change model.

Taken together, our findings show that patient-centered care, depends on both individual competencies and supportive conditions. This interplay becomes clearer when viewed through the COM-B model [34]. This model explains behavior change through the interaction of Capability, Opportunity, and Motivation (COM) leading to Behavior (B). This model is used in implementation science, if behavioral change is needed. In this framework, establishing a shared vision enhances motivation, both intrinsically – personal commitment and professional identity- and extrinsically – alignment with organizational goals. This motivation can enhance Capability, as HCPs develop the necessary skills, knowledge, and confidence to adapt to new expectations, and this creates a learning culture. Additionally, creating an environment that fosters collaboration and provides necessary resources, increases opportunity, making it easier for HCPs to enact desired behaviors. Fig 1 illustrates how the identified themes from our study map onto the COM-B components.

### Strengths and weaknesses

One of the strengths of this study is its qualitative nature, where the combination of FGs and interviews gives a better understanding of the participants’ perspectives and experiences than other research methods. The combination of disciplines led to interesting discussions. The interviews were used to validate the results from the FGs and gave more depth and exploration of personal experiences. All steps of analysis were performed by two researchers (LD, LT), with a senior researcher (EdG) for guidance. This improved the interview quality during the study and the analysis of the data, as the senior researcher’s expertise ensured a more thorough and nuanced interpretation of the findings. Results were discussed within the entire inter-professional team.

A limitation is the professional relationship between interviewers and interviewees. While this facilitated initiating interviews and building rapport, it may have hindered deeper exploration of personal or sensitive topics, potentially leading to socially desirable responses. Interviewers attempted to address this by probing further into the topics.

Additionally, the interviewers’ initial lack of experience affected the quality of the early interviews, reflected in a larger-than-anticipated sample size. However, through reflection and guidance from experienced interviewers, their skills improved over the course of the study. As a result, later interviews were more in-depth and focused on key areas identified earlier, ultimately yielding higher-quality data.

This study provides insights into barriers and facilitators in end-of-life communication with patients with PF, considering both its strengths and limitations.

### Implications for practice

Combining the COM-B model with the results of this study supports the development of implementation strategies necessary to implement palliative care in clinical PF care. The implementation strategies should include establishing a shared vision on palliative care, encouraging HCPs to address not only physical but also psychological, social, and existential dimensions of patients’ lives. Clear guidelines and agreement in roles and responsibilities are also essential.

Improving end-of-life communication requires fostering a learning culture that promotes continuous professional development and supports HCPs. Education, training, and on-the-job coaching—guided by implementation champions—can help strengthen the professional competencies of all members of the multiprofessional team. All strategies are equally important in supporting behavior change and successful implementation of palliative care [35]. Management and champions must actively stimulate HCPs and facilitate their growth.

Further research should explore patients’ perspective on barriers and facilitators in conversations exploring values, preferences, and needs for end-of-life care. Additionally, gaining deeper insights into effective learning strategies for palliative care among HCPs in centers of excellence for rare diseases could improve implementation efforts.

## Conclusion

In conclusion, this study underscores the need for a shared vision on palliative care, a supportive learning environment, and recognition of the individual professional’s role in initiating conversations exploring values, preferences, and needs for end-of-life care for patients with PF. Organizational measures should support behavioral change. Interventions such as structured training, coaching, and team-reflection, can support HCPs in developing the necessary skills and confidence, strengthening a patient-centered approach to palliative care.

## Supporting information

S1 Appendix Interview guide [36]. (DOCX)

## Abbreviations

JH: Janneke Haverkamp
LD: Lea M. Dijksman
LT: Lian Trapman
JG: Jan. C. Grutters
ST: Saskia. C.C.M. Teunissen
EdG: Everlien de Graaf.

## References

[1] Lancaster L, Crestani B, Hernandez P, Inoue Y, Wachtlin D, Loaiza L, et al. Safety and survival data in patients with idiopathic pulmonary fibrosis treated with nintedanib: pooled data from six clinical trials. BMJ Open Respir Res. 2019;6(1):e000397. doi: 10.1136/bmjresp-2018-000397 31179001 PMC6530503

[2] Margaritopoulos GA, Trachalaki A, Wells AU, Vasarmidi E, Bibaki E, Papastratigakis G, et al. Pirfenidone improves survival in IPF: results from a real-life study. BMC Pulm Med. 2018;18(1):177. doi: 10.1186/s12890-018-0736-z 30470213 PMC6251092

[3] Fernández Pérez ER, Daniels CE, Schroeder DR, St Sauver J, Hartman TE, Bartholmai BJ, et al. Incidence, prevalence, and clinical course of idiopathic pulmonary fibrosis: a population-based study. Chest. 2010;137(1):129–37. doi: 10.1378/chest.09-1002 19749005 PMC2803118

[4] Richeldi L, du Bois RM, Raghu G, Azuma A, Brown KK, Costabel U, et al. Efficacy and safety of nintedanib in idiopathic pulmonary fibrosis. N Engl J Med. 2014;370(22):2071–82. doi: 10.1056/NEJMoa1402584 24836310

[5] Crestani B, Huggins JT, Kaye M, Costabel U, Glaspole I, Ogura T, et al. Long-term safety and tolerability of nintedanib in patients with idiopathic pulmonary fibrosis: results from the open-label extension study, INPULSIS-ON. Lancet Respir Med. 2019;7(1):60–8. doi: 10.1016/S2213-2600(18)30339-4 30224318

[6] World Health Organisation. WHO Definition of Palliative Care. https://www.who.int/cancer/palliative/definition/en/

[7] Janssen DJA, Bajwah S, Boon MH, Coleman C, Currow DC, Devillers A, et al. European Respiratory Society clinical practice guideline: palliative care for people with COPD or interstitial lung disease. Eur Respir J. 2023;62(2):2202014. doi: 10.1183/13993003.02014-2022 37290789

[8] IKNL/Palliactief. Netherlands Quality Framework for Palliative Care. 2017.

[9] Rietjens JAC, Sudore RL, Connolly M, Van Delden JJ, Drickamer MA, Droger M. Definition and recommendations for advance care planning: an international consensus supported by the European Association for Palliative Care. The Lancet Oncology. 2017. 10.1016/S1470-2045(17)30582-X 28884703

[10] Zwakman M, Jabbarian LJ, van Delden J, van der Heide A, Korfage IJ, Pollock K, et al. Advance care planning: A systematic review about experiences of patients with a life-threatening or life-limiting illness. Palliat Med. 2018;32(8):1305–21. doi: 10.1177/0269216318784474 29956558 PMC6088519

[11] Brinkman-Stoppelenburg A, Rietjens JAC, van der Heide A. The effects of advance care planning on end-of-life care: a systematic review. Palliat Med. 2014;28(8):1000–25. doi: 10.1177/0269216314526272 24651708

[12] de Vries S, Verhoef M-J, Vervoort SCJM, van der Linden YM, Teunissen SCCM, de Graaf E. Barriers and facilitators that hospital clinicians perceive to discuss the personal values, wishes, and needs of patients in palliative care: a mixed-methods systematic review. Palliat Care Soc Pract. 2023;17:26323524231212510. doi: 10.1177/26323524231212510 38044932 PMC10693227

[13] Mc Veigh C, Reid J, Larkin P, Porter S, Hudson P. The experience of palliative care service provision for people with non-malignant respiratory disease and their family carers: An all-Ireland qualitative study. J Adv Nurs. 2018;74(2):383–94. doi: 10.1111/jan.13453 28910509

[14] Gersten RA, Seth B, Arellano L, Shore J, O’Hare L, Patel N, et al. Provider Perspectives on and Access to Palliative Care for Patients With Interstitial Lung Disease. Chest. 2022;162(2):375–84. doi: 10.1016/j.chest.2022.03.009 35305969 PMC9633804

[15] Bajwah S, Koffman J, Higginson IJ, Ross JR, Wells AU, Birring SS. I wish I knew more… the end-of-life planning and information needs for end-stage fibrotic interstitial lung disease: views of patients, carers and health professionals. BMJ Support Palliat Care. 2013;3(1):84–90. 10.1136/bmjspcare-2012-000263 24644332

[16] Lindell KO, Kavalieratos D, Gibson KF, Tycon L, Rosenzweig M. The palliative care needs of patients with idiopathic pulmonary fibrosis: A qualitative study of patients and family caregivers. Heart Lung. 2017;46(1):24–9. doi: 10.1016/j.hrtlng.2016.10.002 27871724 PMC5485906

[17] Bramhill C, Langan D, Mulryan H, Eustace-Cook J, Russell A-M, Brady A-M. A scoping review of the unmet needs of patients diagnosed with idiopathic pulmonary fibrosis (IPF). PLoS One. 2024;19(2):e0297832. doi: 10.1371/journal.pone.0297832 38354191 PMC10866483

[18] Mortelmans D. Handboek kwalitatieve onderzoeksmethoden. 2nd ed. Leuven: Acco uitgeverij. 2007.

[19] O’Brien BC, Harris IB, Beckman TJ, Reed DA, Cook DA. Standards for reporting qualitative research: a synthesis of recommendations. Acad Med. 2014;89(9):1245–51. doi: 10.1097/ACM.0000000000000388 24979285

[20] Fuller D. Part of the action, or ‘going native’? Learning to cope with the ‘politics of integration’. Area. 1999;31(3):221–7. doi: 10.1111/j.1475-4762.1999.tb00086.x

[21] World Medical Association. WMA Declaration of Helsinki: ethical principles for medical research involving human subjects. 1974;353(1):1418–9.

[22] Organization WH. Handbook for Good Clinical Practice (GCP). 2002.

[23] European Parliament, Council of the European Union. Regulation (EU) 2016/679 of the European Parliament and of the Council of 27 April 2016 on the protection of natural persons with regard to the processing of personal data and on the free movement of such data, and repealing Directive 95/46/EC. Off J Eur Union. 2016:1–88.

[24] Braun V, Clarke V. Successful Qualitative Research: A Practical Guide for Beginners. 1st ed. London: Sage LTD. 2013.

[25] UNESCO Institute for Statistics. International Standard Classification of Education. Montreal, Quebec, Canada: UNESCO Institute for Statistics. 2011.

[26] Vermandere M, Warmenhoven F, Van Severen E, De Lepeleire J, Aertgeerts B. The Ars Moriendi Model for Spiritual Assessment: A Mixed-Methods Evaluation. Oncol Nurs Forum. 2015;42(4):E294-301. doi: 10.1188/15.ONF.294-301 26148311

[27] Slåtten T, Mutonyi BR, Lien G. Does organizational vision really matter? An empirical examination of factors related to organizational vision integration among hospital employees. BMC Health Serv Res. 2021;21(1):483. doi: 10.1186/s12913-021-06503-3 34016114 PMC8139162

[28] Martin J, Mccormack B, Fitzsimons D, Spirig R. The importance of inspiring a shared vision. Int Pract Dev J. 2014;4(2):1–15.

[29] Mlambo M, Silén C, McGrath C. Lifelong learning and nurses’ continuing professional development, a metasynthesis of the literature. BMC Nurs. 2021;20(1):62. doi: 10.1186/s12912-021-00579-2 33853599 PMC8045269

[30] Reeves S, Perrier L, Goldman J, Freeth D, Zwarenstein M. Interprofessional education: effects on professional practice and healthcare outcomes. Cochrane Database Syst Rev. 2013;3:1465–858. 10.1002/14651858.CD002213.pub3 PMC6513239 23543515

[31] Drude KP, Maheu M, Hilty DM. Continuing Professional Development: Reflections on a Lifelong Learning Process. Psychiatr Clin North Am. 2019;42(3):447–61. doi: 10.1016/j.psc.2019.05.002 31358124

[32] Kuipers SJ, Nieboer AP, Cramm JM. Easier Said Than Done: Healthcare Professionals’ Barriers to the Provision of Patient-Centered Primary Care to Patients with Multimorbidity. Int J Environ Res Public Health. 2021;18(11):6057. doi: 10.3390/ijerph18116057 34199866 PMC8200113

[33] Frakking T, Michaels S, Orbell-Smith J, Le Ray L. Framework for patient, family-centred care within an Australian Community Hospital: development and description. BMJ Open Qual. 2020;9(2):e000823. doi: 10.1136/bmjoq-2019-000823 32354755 PMC7213886

[34] Michie S, van Stralen MM, West R. The behaviour change wheel: a new method for characterising and designing behaviour change interventions. Implement Sci. 2011;6:42. doi: 10.1186/1748-5908-6-42 21513547 PMC3096582

[35] Nilsen P. Implementation science, theory and application. New York; 2024.

[36] Evers J. Kwalitatief interviewen: kunst én kunde. 2 ed. Amsterdam: Boom uitgevers. 2015.

