# Supplementary material for: Perspective of healthcare professionals on barriers and facilitators in exploring end-of-life care preferences of patients with pulmonary fibrosis: A qualitative study
Source: PLoS One. 2025 Dec 12;20(12):e0338624. doi: 10.1371/journal.pone.0338624 (PMC12700373; doi:10.1371/journal.pone.0338624)
Supplement: S1 Appendix — (DOCX) [file pone.0338624.s001.docx]

# Attachment 1: Interview guides

| **Interview guide focus groups** | |
| --- | --- |
| **Focus group 1:** | |
| Method: | Discussion following the river-method. [36] |
| General questions: | - What is palliative care for you? - How do you feel about the palliative care nowadays in our clinic; (what are your experiences with this?) - What are the barriers for conversation about the end of life? - What are the facilitators for conversation about the end of life? - How do you see video-consulting in palliative care? - When do you feel you have a relationship of trust with your patient? - When does the palliative phase starts? |
| **Focus group 2** | |
| Method: | ‘zang- en klaag muur’ / ‘Sing and complain wall’ |
| General question: | Write down on the ‘singing’ and ‘complaining’ wall all aspects of palliative care in your clinic which you want to sing or complain about. |
|  | Plenary discussion |
| **Focus group 3** | |
| Method | Visualisation/dreaming with discussion afterwards |
| General question: | Without boundaries, how would you like the palliative care in your clinic to be? |
|  | Plenary discussion |

| **Interview guide individual interviews** | |
| --- | --- |
| Method | River-method [36] |
| Techniques used | - Building rapport - Summarization - Request specific examples - Employing strategic silences - Member checking during |
| General question | - What is palliative care for you? - What is your experience with palliative care? - What is your experience with end of life conversations? - Could you tell me how you feel about talking about end of life care? - Which tools do you already use in these conversations? - What are your needs/preconditions to start this conversation? - How do you feel about video-consulting in palliative care/end of life care? - How do you think about communication between healthcare professionals in end of life care? - How is the division of tasks (tasks, responsibilities and authorisation)and how would you ideally see this? - At which moment you think this conversation should be started? |
